# GeCKO: user-friendly workflows for genotyping complex genomes using target enrichment capture. A use case on the large tetraploid durum wheat genome

**DOI:** 10.1186/s13007-024-01210-6

**Published:** 2024-07-13

**Authors:** Morgane Ardisson, Johanna Girodolle, Stéphane De Mita, Pierre Roumet, Vincent Ranwez

**Affiliations:** 1 UMR AGAP Institut, Univ Montpellier, CIRAD, INRAE, Institut Agro, 34398 Montpellier, France; 2 INRAE, CIRAD, Institut Agro, IRD, PHIM, Université Montpellier, Montpellier, France

**Keywords:** Complex genome, Domestication, Durum wheat, FAIR principles, Genotyping, Target enrichment capture, Workflows

## Abstract

**Background:**

Genotyping of individuals plays a pivotal role in various biological analyses, with technology choice influenced by multiple factors including genomic constraints, number of targeted loci and individuals, cost considerations, and the ease of sample preparation and data processing. Target enrichment capture of specific polymorphic regions has emerged as a flexible and cost-effective genomic reduction method for genotyping, especially adapted to the case of very large genomes. However, this approach necessitates complex bioinformatics treatment to extract genotyping data from raw reads. Existing workflows predominantly cater to phylogenetic inference, leaving a gap in user-friendly tools for genotyping analysis based on capture methods. In response to these challenges, we have developed GeCKO (Genotyping Complexity Knocked-Out). To assess the effectiveness of combining target enrichment capture with GeCKO, we conducted a case study on durum wheat domestication history, involving sequencing, processing, and analyzing variants in four relevant durum wheat groups.

**Results:**

GeCKO encompasses four distinct workflows, each designed for specific steps of genomic data processing: (i) read demultiplexing and trimming for data cleaning, (ii) read mapping to align sequences to a reference genome, (iii) variant calling to identify genetic variants, and (iv) variant filtering. Each workflow in GeCKO can be easily configured and is executable across diverse computational environments. The workflows generate comprehensive HTML reports including key summary statistics and illustrative graphs, ensuring traceable, reproducible results and facilitating straightforward quality assessment. A specific innovation within GeCKO is its 'targeted remapping' feature, specifically designed for efficient treatment of targeted enrichment capture data. This process consists of extracting reads mapped to the targeted regions, constructing a smaller sub-reference genome, and remapping the reads to this sub-reference, thereby enhancing the efficiency of subsequent steps.

**Conclusions:**

The case study results showed the expected intra-group diversity and inter-group differentiation levels, confirming the method's effectiveness for genotyping and analyzing genetic diversity in species with complex genomes. GeCKO streamlined the data processing, significantly improving computational performance and efficiency. The targeted remapping enabled straightforward SNP calling in durum wheat, a task otherwise complicated by the species' large genome size. This illustrates its potential applications in various biological research contexts.

## Introduction

Over the past decade, a wide range of genotyping technologies have emerged to expedite and enhance the efficacy of large-scale genotyping projects. These technologies are particularly crucial in the field of agronomy, where they are accelerating plant breeding programs. Among these advances, the advent of Next-Generation Sequencing (NGS) stands out for its role in democratizing access to sequence data.

While whole-genome sequencing is increasingly common for species with small genomes such as Arabidopsis [1], or those of high medical or agronomical importance such as humans [2] and rice [3], it presents significant challenges for large-genome species. For instance, the genomes of key agronomic species like bread and durum wheats are more than 3 and 5 times larger than the human genome with respectively 10.5 and 17 GB [4], making whole-genome resequencing impractical without substantial sequencing and computational resources. Meanwhile, for various applications such as phylogenetic inference or population genotyping, whole-genome coverage often proves to be superfluous [5–7].

Considering these constraints, various methods serve as alternatives to whole-genome sequencing, each adapted to specific needs and sample sizes. First among these are allele-specific assays, such as DNA chips, which offer the advantage of speed and simplicity. These are particularly useful for large-scale studies aiming to explore genetic variation across whole populations or for rapid screening of specific mutations. However, they necessitate prior knowledge of the expected allelic forms in the individuals being genotyped. Alternatively, genomic reduction strategies combined with NGS, like target enrichment capture, present a cost-effective and customizable solution for high-throughput sequencing of specified regions of the genome [8]. Although these methods generally require more sample preparation and resources, they enable a more in-depth examination of genomic regions that have been purposefully selected for the study, including the detection of polymorphisms beyond biallelic Single Nucleotide Polymorphisms (SNPs). Given these strengths, which are particularly relevant in the context of population genetics, this paper will focus on the data processing aspects of target enrichment capture.

In essence, this method involves three main steps. First, a predetermined set of target loci is selected, and 'baits' -short sequences specific to these regions- are synthesized. Second, DNA is extracted, fragmented, and amplified from each sample to construct genomic libraries. Finally, sequence hybridization between the libraries and baits is performed to selectively capture and sequence the fragments of interest. Because the targeted regions often constitute a small fraction of the entire genome, this approach significantly enhances the efficiency of the sequencing process. Specifically, what would have been a single sequencing run yielding poor coverage of an individual's entire genome can now produce high-coverage data for the targeted regions across multiple individuals, discriminated by barcode identifiers.

Target enrichment capture proves especially adept at addressing complex evolutionary questions such as domestication, particularly when concerning large-genome species. It offers an unbiased lens for detecting polymorphic variations [9], a great quality for documenting diversity patterns along both spatial and temporal gradients.

Durum wheat, characterized by its extensive and complex genome as well as its rich history of domestication, is thus a perfect illustration of the benefit of using target enrichment capture. This species has experienced multiple demographic and selective events since the onset of its domestication in the Fertile Crescent. The first significant shift transpired approximately 12,000 years ago, coinciding with the societal transition from hunter-gatherer lifestyles to settled agricultural communities. One of the key phenotypic markers for this initial stage of domestication is the development of a solid rachis [10], indicative of the transformation from wild emmer (*Triticum turgidum* subsp. *dicoccoides*) to cultivated emmer (*Triticum turgidum* subsp. *dicoccum*). A subsequent mutation, occurring around 5000 years ago, gave rise to free-threshing forms of wheat. These variants gradually supplanted their hulled counterparts, primarily due to their ease in post-harvest processing [11]. Included among these free-threshing forms is *T. t. durum*, which is currently a staple in the production of semolina and pasta. Most recently, the Green Revolution of the 1960s and 1970s marked another pivotal transition, as traditional ‘landraces’ were replaced by short-stature ‘elite’ varieties to mitigate lodging risks associated with intensive fertilizer application.

The recent sequencing of the *T. turgidum* genomes of a wild accession (Zavitan) and an elite cultivar (Svevo) has provided baseline reference points for studying genetic diversity throughout the domestication process [4, 12]. However, due to the technological, human, and financial resources required to sequence additional durum wheat genomes, limitations exist in further advancing this area of study [13–15]. This is particularly concerning when studying wild accessions, whose unique polymorphisms may be overlooked if genotyping relies solely on DNA chips designed from cultivated accessions, thereby underestimating their genetic diversity. Target enrichment capture advantageously addresses such issues, but the hurdle of processing and interpreting the resulting data has limited its broader application.

Despite the advantages conferred by target enrichment capture, the transition from raw read datasets to a filtered set of SNPs or to aligned orthologous sequences remains a challenging and time-consuming endeavour. This involves the sequential use of multiple software tools, adding layers of complexity that compromise the reproducibility of analyses—an essential component for both scientific credibility and research efficiency [16]. Indeed, adopting a FAIR data approach –making data Findable, Accessible, Interoperable, and Reusable—aligns the research process with best practices. One effective way to address these issues is through the utilization of reproducible workflows. While existing workflows like SECAPR [17], HybPiper [18], and PHYLUCE [19] are well-suited for phylogenetic studies, they fall short in addressing challenges specific to population or quantitative genetics—i.e. a variant calling step-, and lack specific features tailored for target enrichment capture data.

This creates a gap between the promise of this technology and its practical accessibility for biologists aiming to process data from a large number of genotypes in species with complex genomes.

To bridge this gap, we have developed GeCKO—a toolkit comprising four modular workflows, designed to process raw multiplexed reads into a filtered VCF file. GeCKO is especially suited for supporting target enrichment capture-based genotyping of organisms with very large genomes. Hosted on GitHub alongside detailed documentation, each workflow aims to facilitate reproducible analyses, automatically generate comprehensive reports, and strike a balance between user-friendliness, performance, and flexibility. To illustrate GeCKO's capabilities, we applied it to an original dataset of durum wheat, a large-genome species, generated using target enrichment capture methods, aiming to assess how allele diversity has evolved during the domestication process. The subsequent sections will detail GeCKO’s workflows and its effectiveness and efficiency in managing this specific dataset and providing files suitable for downstream analysis. This use case illustrates the potential of GeCKO to promote the wider application of target enrichment capture in complex genomic studies.

## Methods

### Implementation

#### Using Conda, Snakemake and Github to ensure portability, scalability, and reproducibility

Workflow managers allow easy leverage of the computation power of HPC clusters and cloud environments by automating the parallelization process (scalability). By separating environment configuration from bioinformatics analysis per se, workflow managers ease switching from one HPC environment to another (portability), while ensuring that the analysis can be rerun using the exact same tools (reproducibility). Using a workflow manager has many advantages over writing workflows from scratch in generic scripting languages like bash, Perl, or Python. Several workflow managers, each with their pros and cons, are available (for a recent overview, interested readers could refer to [20]). Here we choose to rely on Snakemake to develop our GeCKO workflows [21, 22].

Snakemake, with over a decade of development, is widely adopted in the bioinformatics community. It facilitates workflow modularity and the easy creation of custom environments equipped with the necessary bioinformatic tools through Mamba [23], a faster and more efficient drop-in replacement for the Conda package manager [24]. Additionally, Snakemake is grounded in Python, a language widely utilized in science and particularly user-friendly and adaptable for population geneticists.

While Snakemake also supports Singularity and Docker containers for creating controlled environments, using Mamba seemed to be simpler and more flexible in our case. Snakemake's ability to automate Conda environment creation circumvents the need for container recipes and the challenges associated with container use on HPC clusters, such as non-standard directory structures.

For reproducibility and traceability purposes, our workflows automatically document essential information at each run. This includes the run’s date and time, used configuration files, the GitHub commit ID of the current workflow version, and the creation timestamps of output files, all logged in a workflow_info.txt file. Additionally, if the workflow is rerun (e.g., to generate missing files), the information from the latest run is appended to the end of this file. This ensures that users can trace all runs that may have contributed to the generation of any output files, thereby enhancing the traceability and transparency of the research process.

#### Using MultiQC to produce informative task reports

Genome-scale data analysis for genotyping numerous individuals generates hundreds of files at each step, making monitoring the overall process challenging. Although workflow managers aid in parallelizing tasks, detecting issues early in the process is often difficult, with problems typically only becoming apparent after the workflow's completion. Traditionally, intermediary quality checks depend on tedious, ad-hoc scripts. MultiQC [25] addresses this by automating the generation of visual HTML reports. It parses output files from various bioinformatics tools, creating informative summaries that are then integrated into a comprehensive report. This report tracks individual sample names and highlights potential issues, such as individuals with very low read coverage that may require re-sequencing, low mapping percentages indicative of potential inter-species contamination, identification of loci not correctly captured by the designed baits, or a drastic drop in the number of SNPs after certain filtering steps. In the GeCKO workflow, MultiQC’s capabilities are leveraged to produce detailed reports, enabling prompt identification of these issues.

#### A homogeneous architecture for the four workflows of GeCKO

GeCKO comprises four main workflows: DataCleaning, ReadMapping, VariantCalling, and VcfFiltering. Each workflow is configured through three distinct files: one for specifying software release versions, another for cluster-related details (like partition or queue names, memory and CPUs), and a third for workflow-specific parameters (such as input file paths and bioinformatics tool options). The first parameter file (“conda_tools.yml”) can be found in each GeCKO workflow’s WORKFLOW/ENV subfolder. It relies on Mamba to select the software releases to be used, as well as their dependencies. The second file is a cluster configuration file, and needs to be modified to fit the user’s data and HPC cluster environment. GeCKO offers configuration examples for both Slurm [26] and SGE [27] job schedulers, allowing users to specify partitions (for Slurm) or queues (for SGE) and adjust memory requirements for individual tasks. Slurm and SGE templates for this file are provided with each GeCKO workflow’s example dataset (e.g., “DC_CLUSTER_PROFILE_SLURM/config.yaml”).

The final configuration file specifies parameters directly related to data analysis, such as input file paths and options for the bioinformatics tools used in the workflow. For each workflow, GeCKO provides at least one template configuration file (e.g., “config_DataCleaning.yml”), which users can easily customize by replacing default values with their specific parameters. Detailed information about the available options can be found in the GeCKO documentation on GitHub. The configuration files use the YAML format, which offers a user-friendly way to assign values to parameters. An example excerpt from the “config_DataCleaning.yml” file might include:
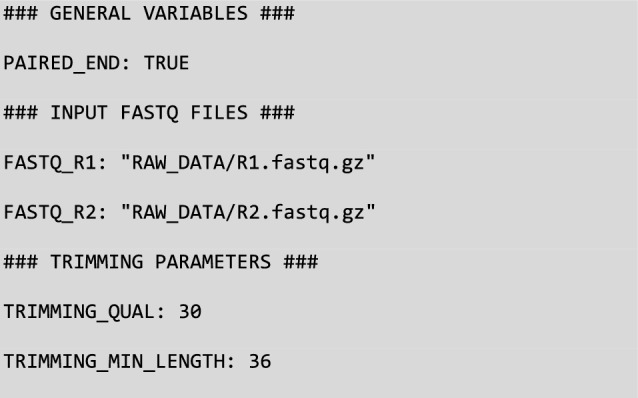


The output files of every GeCKO workflow are grouped in a folder named after the selected workflow (e.g., “DATA_CLEANING”), created at the root of the general output folder “WORKFLOWS_OUTPUTS”. When default paths and file names are used, GeCKO workflows can automatically locate the files they need from previous analysis steps, alleviating the need to specify each file path manually (though this option remains available).

#### Bioinformatics tools

Each workflow seamlessly integrates various data processing steps, employing an array of well-established bioinformatics tools.

In the DataCleaning workflow, Cutadapt [28] (v3.5) is used for both demultiplexing reads—assigning each read to its original sample using barcodes incorporated during library preparation—and for trimming tasks, such as removing adapter sequences and low-quality nucleotides, and discarding excessively short reads post-trimming. Additionally, FastQC [29] (v0.11.9) is used to monitor read quality throughout these stages.

The ReadMapping workflow offers four different mappers: bwa (v0.7.17, mem function [30]), bwa-mem2 [31] (v2.2.1, mem function), Bowtie 2 [32] (v2.4.5), and Minimap2 [33] (v2.24), allowing users to align reads against a reference according to their specific needs to produce BAM files. Subsequent processing and filtering of the mapped reads are carried out using Samtools [34] (v1.14), Picard [35] (v2.26.10), and bedtools [36] (v2.30.0).

The VariantCalling workflow is executed using GATK [37] (v4.2.5.0), chaining the three functions HaplotypeCaller, GenomicsDBImport, and GenotypeGVCFs to detect polymorphisms among the provided BAM files.

In the VcfFiltering workflow, BCFtools [38] (v1.15.1) and the EggLib Python library [39] (v3.1.0) are used to respectively filter SNPs based on various criteria and to compute population genetic indicators such as Nei’s genetic diversity (He) or the inbreeding coefficient (Fis).

In the DataCleaning, ReadMapping and VcfFiltering workflows, MultiQC (v1.11) is used to aggregate and report data quality metrics, as previously described.

#### Installing and executing GeCKO workflows

To install GeCKO, one simply needs to clone the GitHub project (https://github.com/GE2POP/GeCKO) onto a computer or HPC cluster with Conda available (release 22.9.0 or higher). GeCKO is compatible with Linux and macOS operating systems, and can also be run on Windows through the Windows Subsystem for Linux (WSL). While there are no minimum system requirements for installation, the available computational resources—such as RAM and CPU capacity—will greatly influence the dataset size that can be handled effectively. This is dependent on both the dataset’s number of sequences and the size of the reference genome. For instance, we executed all four workflows on our test dataset available on GitHub—comprising 100,000 read pairs to be mapped to a 50 Mb reference—on a basic laptop (Intel Core i5-3340 M CPU, 8 GB RAM), completing the process in 40 min. In a more resource-intensive scenario, the same workflows applied to the durum wheat dataset described in our use-case (436,000,000 read pairs with a 10.5 Gb reference genome) took 2.6 days to complete on a high-performance computational cluster (requiring up to 27 GB RAM for some of the VariantCalling steps). Detailed installation instructions are in the GitHub repository's readme file, which also includes sample datasets and configuration templates for easy setup. A convenient launcher script, runGeCKO.sh, is also provided. To help prevent potential compatibility issues, it is recommended to use the launcher to create a Conda environment that includes Snakemake (currently version 7.32.4), Mamba (currently version 1.4.9) and their dependencies with:
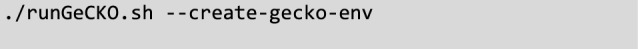


To execute a workflow, the Conda environment should first be activated with:
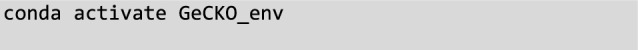


Then, the launcher can be invoked, using the “–workflow” argument to specify the workflow’s name (i.e., DataCleaning, ReadMapping, VariantCalling or VcfFiltering). By default, the script will look for the config_DataCleaning.yml configuration file in a CONFIG folder, but you can specify a different file and path using the script argument “–config-file”. The cluster configuration file is passed with the “–cluster-profile” argument by providing the path to the directory containing the config.yaml file (e.g. “DC_CLUSTER_PROFILE_SLURM”). The script not only executes workflows but also offers Snakemake features for testing workflow consistency (–dryrun), generating usage reports including CPU usage per task (–report), or creating workflow diagrams (–diagram). To execute the DataCleaning workflow on a Slurm-based HPC environment with up to 100 concurrent jobs, the command would be:
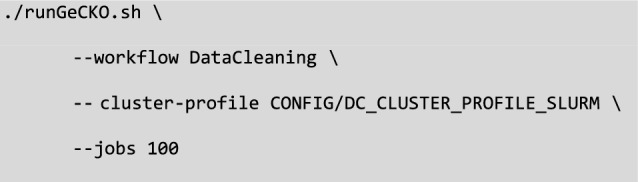


### Use case

In this section, we detail the methodology used to acquire sequence data, forming a dataset suitable for our use case. The subsequent analysis of this dataset using the GeCKO workflows is described in the Results section, illustrating its practical application.

#### Biological material

**Fig. 1:**
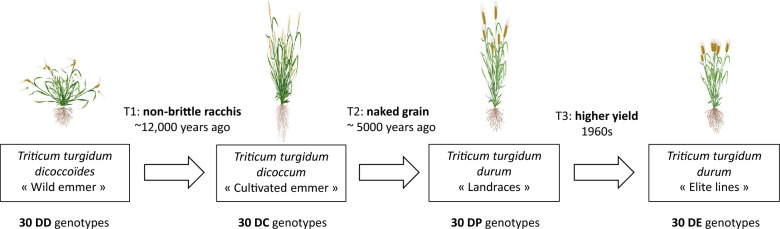
Biological sampling used to study the domestication process of durum wheat through four sub-populations (DD, DC, DP, and DE)

In this study, we focus on 120 accessions from the three major subspecies involved in the four transition steps of the durum wheat domestication (Fig. 1): the wild form *T. turgidum dicoccoides* (n = 30, denoted DD), the first domesticated form with a solid rachis, *T. turgidum dicoccum* (n = 30, DC), and the non-hulled cultivated form, *T. turgidum durum* (n = 60). The fourth step of domestication occurred within the latter subspecies, which is further divided into two groups based on whether the varieties originated in the pre- or post-Green Revolution period. The first group consists of “Landraces” (n = 30, DP), which are lines derived from local varieties, and the second group of “elite” varieties (n = 30, DE) registered in Europe post-Green Revolution (1970–1990). These 120 accessions were selected from a 314-accession collection [13] to maximize the genetic diversity within each group, using the MSTRAT software [40].

#### From biological samples to raw reads

For each of the 120 genotypes, genomic DNA was extracted from 50 mg of plant material. After grinding in nitrogen and cell lysis (extraction buffer with SDS, CTAB), the DNA was purified on a KingFisher™ Flex Purification System (Perkin-Elmer Chemagen metal and magnetic beads). The quality of the extracted DNA was assessed through 1.5% agarose gel electrophoresis and spectrophotometric assay (absorbance at 260/280 nm and 260/230 nm—SPARK10M™ TECAN), while DNA quantity was estimated using a spectrofluometric assay (Hoescht 33258-InfiniteM200™ TECAN). Genomic libraries were prepared following the protocol presented by Holtz et al. [9], specifically tailored for subsequent target enrichment capture, with two key modifications: (i) the ligation of barcodes on both ends of the DNA fragments to eliminate chimeric molecules formed during PCR; and (ii) the multiplexing of libraries prior to targeted enrichment, ensuring a uniform number of reads per genotype through precise quantification.

Targeted enrichment capture allows to selectively isolate and sequence specific regions of interest from a genome, using probes (baits) that hybridize to those regions. In our case, we utilized the 20,000 baits designed by Holtz et al. targeting 6240 SNPs within coding regions across the durum wheat genome. These baits, each 120 nt long, were synthesized by Arbor Biosciences (https://www.arborbiosci.com/). Following Holtz et al.’s protocol, target enrichment capture was conducted using the SeqCap EZ Hyb and Wash kit (Roche). Given the complexity of the durum wheat genome and the minuscule proportion represented by the targeted regions (about 0.1% of the genome), we optimized the capture process by: (i) carrying out two successive capture phases to enhance hybridization specificity and, consequently, enrichment efficiency, and (ii) using blockers to saturate highly repetitive genomic regions and reduce their interference.

Finally, the captured sequences were amplified and quantified before undergoing paired-end sequencing (2 × 150 bp) on an Illumina Hiseq3000™ high-throughput sequencer. To ensure sufficient coverage of the targeted loci, the libraries from the 120 genotypes were divided into two pools, captured and sequenced independently: DEV_Cap009 (62 genotypes) and DEV_Cap010 (58 genotypes). The sequenced data from each capture experiment were delivered as two compressed fastq files (paired R1 and R2). This forms the dataset that will be processed using the GeCKO workflows, as detailed in the subsequent sections.

## Results

### GeCKO workflows, usage, and key features

**Fig. 2:**
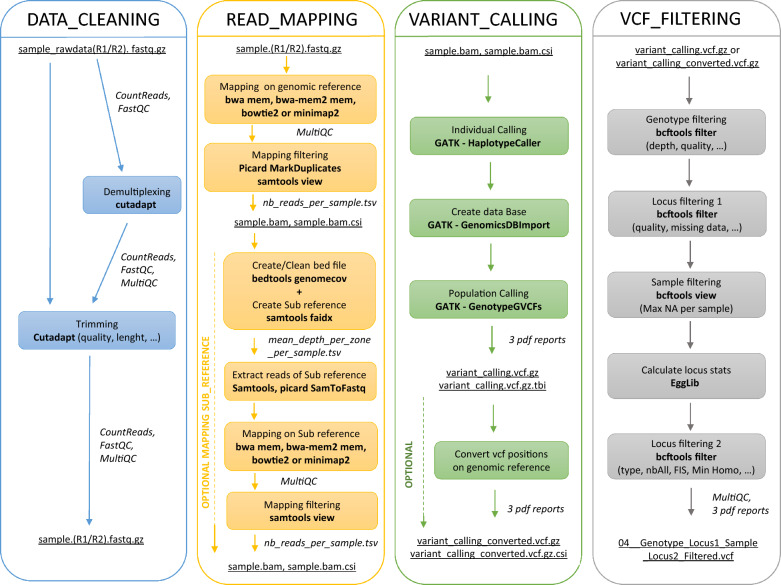
Schematic representation of the four GeCKO workflows

This section provides an overview of the four available workflows, detailing their parameters, inputs and outputs (Fig. 2), and presenting the use case results for illustration.

#### The DataCleaning workflow

##### Purpose and description

This workflow processes raw sequencing data in fastq format to generate a set of 'clean sequences' for each sample. It accommodates both single-end (SE) and paired-end (PE) reads, including multiplexed datasets. For multiplexed data, a tabular file listing each sample’s specific barcode sequence is required. In this case, the workflow will first demultiplex the dataset to produce one (for SE) or two (for PE) fastq file(s) per sample. Reads are then trimmed, regardless of whether demultiplexing was performed.

##### Use case: cleaning multiplexed paired-end reads

**Fig. 3:**
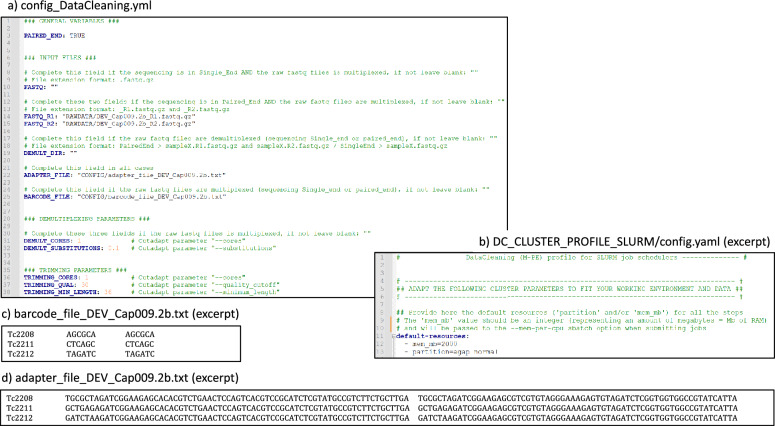
Config and input files of the GeCKO data cleaning workflow. The YAML workflow configuration file (**a**), the cluster configuration file (**b**), and the two tabular files providing the barcodes (**c**) and adapters (**d**) used for sequencing samples through a multiplexed approach

The configuration file config_DataCleaning.yml (Fig. 3a) specifies that our data are paired-end reads (L3), provides the paths to (i) the two fastq files related to DEV_Cap009 capture experiment (L14–15) (ii) the adapter file (L22 and Fig. 3d) and (iii) the barcode file (L25 and Fig. 3c). This parameter file also indicates that our data are multiplexed (since DEMULT_DIR is set to an empty string, L19). Finally, this file allows one to pass parameters to Cutadapt both for the demultiplexing (L31–32) and trimming tasks (L36–38).

In the provided cluster configuration file, DC_CLUSTER_PROFILE_SLURM/config.yaml (Fig. 3b), “mem-mb” is defaulted to 2G (L12), aligning with the workflow’s tasks' minimal resource needs.

**Fig. 4:**
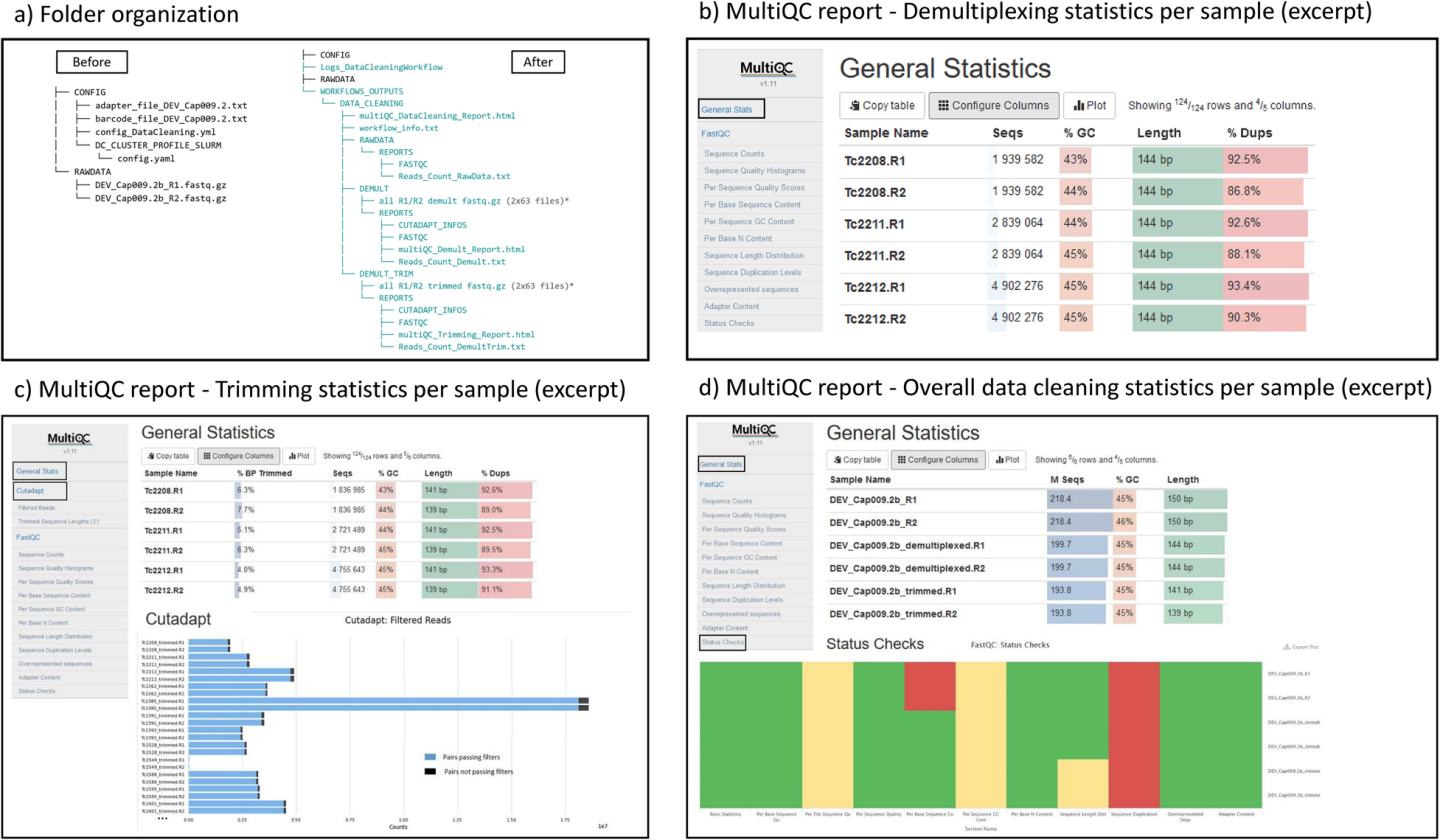
Key outputs of the GeCKO data cleaning workflow for the DEV_Cap009 experiment. The folder organization of output files (**a**) and excerpts of the demultiplexing statistics (**b**) trimming statistics (**c**) and overall cleaning statistics (**d**)

This workflow generates two files at the root of the WORKFLOWS_OUTPUTS/DATA_CLEANING folder: an HTML file providing a synthetic report of the analysis and the “workflow_info.txt” text file. All other output files are organized using one subfolder per key analysis step, i.e., “RAWDATA”, “DEMULT” and “DEMULT_TRIM” (Fig. 4a). In our case, the “DEMULT_TRIM” folder contains 126 fastq.gz files (2 × 62 genotypes + 2 unknown) whereas the “REPORT” section in the “DEMULT” and “DEMULT_TRIM” folders provides key statistics concerning input and output data. These two reports are provided as HTML documents generated by MultiQC and a menu on their left provides immediate access to the various statistics (Fig. 4b–d).

The demultiplexing statistics (Fig. 4b) detail the number of sequences assigned to each sample, along with their average GC content and length. Here, the average read length of 144 bp is consistent with typical Illumina short reads, and, as expected, the number of R1 reads matches the number of R2 reads for each sample. While the GC content is relatively uniform across samples, there is a notable variation in the number of reads—ranging from approximately 2 million read pairs for sample Tc2208 to around 4.9 million for sample Tc2212 in the excerpt shown in the figure. Post-trimming, the output table (Fig. 4c) offers a comprehensive breakdown for each sample (row), including the count of raw reads; the percentage of discarded reads, and the average length and GC content of these trimmed reads. The MultiQC report includes a bar plot contrasting the number of reads retained (in blue) against those removed (in black) for each sample, aiding in the identification of outliers. In this analysis, two outliers were identified: Tc2549 and Tc2385, with the smallest (45,247) and largest (~ 18 million) read pair counts, respectively. These figures significantly deviate from the average count of approximately 3 million. Tc2549, with its abnormally low count of trimmed reads, was excluded from further analysis. Regarding Tc2385, investigations revealed that this was caused by a dosage error in the laboratory during the construction of the multiplexed library.

The overall data cleaning statistics (Fig. 4d) offer an overview of the quality across raw, demultiplexed, and trimmed data for the entire experiment (in this case, DEV_Cap009). Notably, the “Per Base Sequence Content” shows marked improvement post-demultiplexing, indicated by a change in color code from red (raw data) to green (demultiplexed and trimmed data). Initially, all reads are of uniform length, but post-trimming, variations in read lengths emerge, reflected by the ‘Sequence Length Distribution’ report’s warning flag changing from green to yellow. A persistent concern, however, is the 'Sequence Duplications' proportion, which remains flagged as problematic (red) in the FastQC report after trimming. This issue could stem from PCR/optical duplicate artifacts or from an exceptionally high sequencing depth, a plausible scenario when using baits for targeting a minuscule fraction of the genome.

Since we observe a very high proportion of duplicates (> 80%) even among the 45,247 reads of the TC2549 sample, it seems clear that we are dealing with duplicate artifacts, and not with reads obtained by sequencing fragments from a genomic sequence fragmented in the exact same manner multiple times, as is common in GBS analysis (enzymatic fragmentation). A critical concern with PCR duplicates lies in the potential amplification of early-cycle replication errors, leading to duplicated reads containing these inaccuracies. This can mislead variant calling algorithms to consider these errors as genuine polymorphisms, falsely supported by multiple reads. For this reason, it was decided to discard them during the read mapping step. Although this means excluding nearly 90% of the reads, the remaining 10% represents an average of 300,000 reads per sample, which is still a significant amount of data when targeting only 6240 SNPs.

Enrichment capture does not typically result in such high PCR duplicate rates. This was an unanticipated consequence of the extremely large genome size of durum wheat. This is a perfect example of the benefits of having automatically generated and easy to read GeCKO reports, which in this case allowed us to address the issue by revising our laboratory protocols.

#### The ReadMapping workflow

##### Purpose and description

This workflow aligns demultiplexed, trimmed fastq reads (from one or more folders), either single-end or paired-end, to a genomic reference, accommodating the following mapper options: ‘bwa-mem2_mem’, ‘bwa_mem’, ‘bowtie2’ and ‘minimap2’. Users can customize mapping parameters, including options for duplicate removal and Java settings. Post-mapping, the workflow offers optional steps to filter BAM files, such as using Picard’s MarkDuplicates for PCR duplicate removal and samtools view for excluding specific alignments like unmapped, improperly paired, secondary, or supplementary mappings.

Following the initial mapping step, an optional reduction of the initial genomic reference to solely the targeted or covered regions can be performed. The rationale for this genomic reduction is to expedite subsequent steps and is particularly relevant for species with very large genomes. Additionally, some SNP callers impose a maximum chromosome size limit (e.g., 520 Mb for GATK), making it impractical to use the complete genomic reference for these species. The genomic reduction addresses these constraints by creating a smaller reference, which we term a ‘sub-reference.’ This step can be executed using either a set of user-specified targeted genomic regions (with coordinates provided in BED format) or based on the mapping results. In the first case, if the user aims to extract zones that correspond to what their baits are designed to capture, they must first independently identify these targeted zones. This can be done by blasting the bait sequences against the reference genome and extracting the coordinates of these zones. The workflow then automatically merges any potential overlapping regions in the provided BED file. In the second case, GeCKO identifies relevant genomic regions based on read coverage and generates a BED file listing these regions. This process is guided by three user-defined parameters in the config file: BED_MIN_MEAN_COV, BED_MIN_DIST, and BED_MIN_LENGTH. Specifically, GeCKO first identifies all bases with a mean coverage per sample above the provided threshold (BED_MIN_MEAN_COV). It then aggregates bases into continuous regions if they are closer than the specified minimum distance (BED_MIN_DIST). Finally, it removes any regions that are shorter than the minimum length (BED_MIN_LENGTH) after merging. Subsequently, reads mapped to these targeted or covered genomic regions are extracted, converted back into fastq format, and remapped onto the sub-reference. We refer to this process as targeted remapping. To guarantee that reads which were properly paired in the initial mapping do not become improperly paired should they span two distinct regions, the workflow performs zone-by-zone extraction of paired-end reads. This approach ensures that such reads are instead treated as single-end reads during the remapping process. After this second mapping step, filters can be applied to the sub-BAM files using samtools view (e.g. to select reads with high-quality mapping scores).

##### Use case: mapping reads on a large genomic reference

Reads from 119 samples (61 from DEV_Cap009 and 58 from DEV_Cap010) were mapped to the Zavitan reference genome (NCBI assembly GCF_002162155.1).

**Fig. 5:**
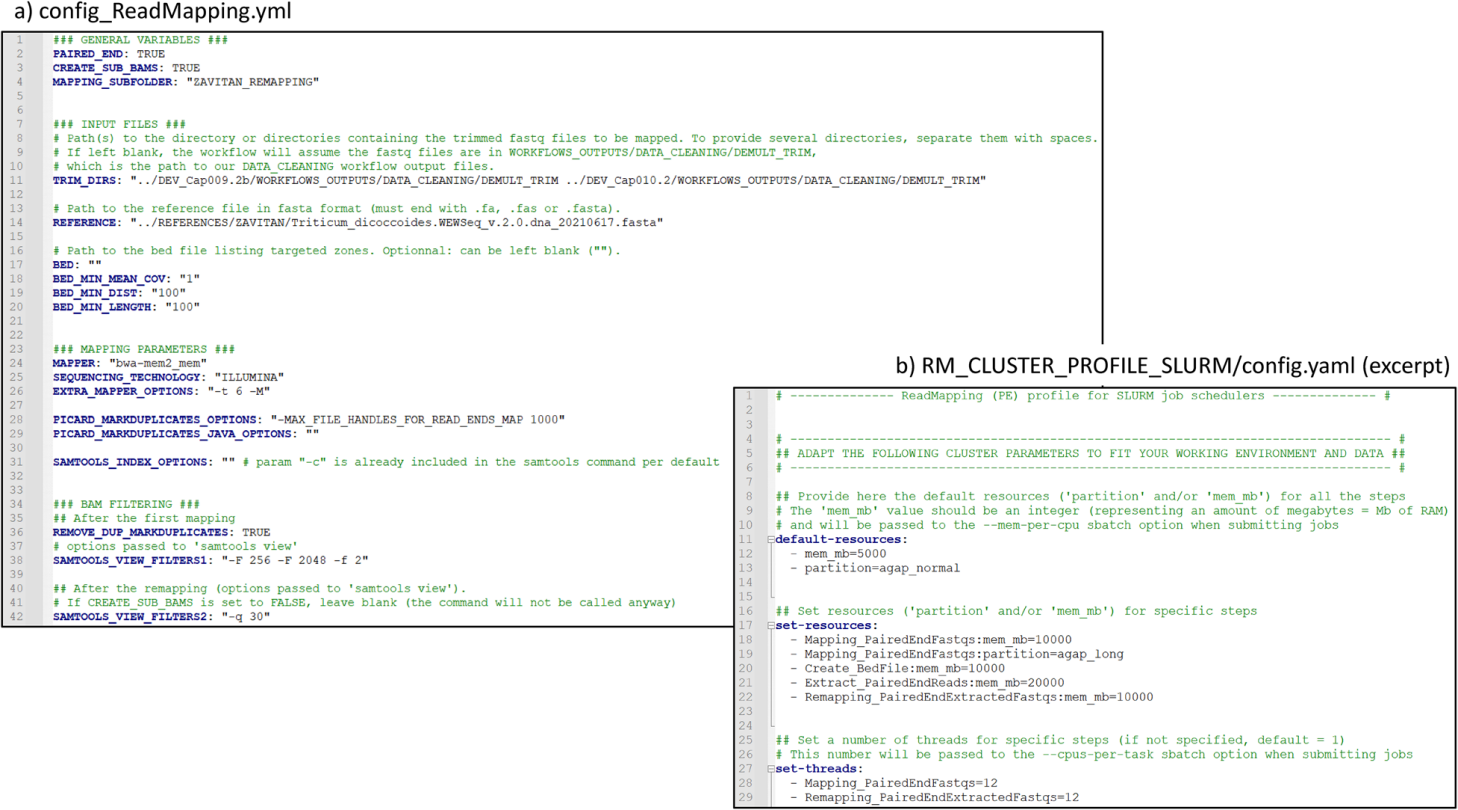
Config files of the GeCKO read mapping workflow. The YAML workflow configuration file (**a**), and the cluster profile file (**b**)

As mapping reads on the complete reference genome of durum wheat is time- and memory-intensive, we adjusted the read mapping step cluster settings accordingly as shown in Fig. 5b. This included increasing memory allocation in the ‘set-resources’ section (mem_mb = 10000, L18) and allowing the jobs to run on a partition designed for durations exceeding 24 h on our Slurm cluster (partition = agap_long, L19). Additionally, the ‘set-threads’ section (L28-29) allowed us to increase the number of CPUs needed for the Mapping_PairedEndFastqs and Remapping_PairedEndExtractedFastqs steps to 12.

As for the config file (Fig. 5a), it allows to specify the paths to the reference genome (Fig. 5a, L14) and the directories containing the fastq files to be mapped (Fig. 5a, L11). Concerning the mapping process, we opted for bwa-mem2 (L24) and tailored the mapper options to our requirements (option EXTRA_MAPPER_OPTIONS, L26). Additionally, we configured specific Picard MarkDuplicates options (L28, 29).

Given the substantial size of the Zavitan reference genome (~ 10.5 Gb), we employed the GeCKO feature CREATE_SUB_BAMS (L3) to extract reads from relevant loci and produce a sub-reference, enhancing the efficiency of subsequent steps. In our case, this sub-reference was constructed by first identifying genome positions with a minimum average of one read per sample (L18). Positions located within 100 bp of each other (L19) were merged into relevant genome regions, and regions smaller than 100bp (L20) were then excluded. This choice allows retaining the vast majority of covered regions while reducing the reference to 10.1 Mb. The resulting sub-reference is provided in FASTA format, accompanied by a BED file detailing the coordinates of these regions within the complete reference genome. After the initial mapping, specific filters were applied to the BAM files. First, duplicates were removed (REMOVE_DUP_MARKDUPLICATES: TRUE, L36). Then, reads were filtered using Samtools view with a first set of filters, provided in SAMTOOLS_VIEW_FILTERS1 ("-F 256 -F 2048 -f 2") at line 38, targeting the exclusion of improperly paired reads, non-primary, and supplementary alignments. In our case, where we opted for a targeted remapping step, this precaution prevents the misclassification of improperly paired reads as singletons during the remapping if only one read of a pair is preserved. Furthermore, in the absence of the primary read (in case it mapped out of the extracted zones), secondary or supplementary alignments could be incorrectly designated as primary during the remapping step. Such misclassifications can lead to the erroneous interpretation of mapping quality, resulting in inaccuracies in downstream analysis and the potential overestimation of certain reads' reliability.

Following the remapping step, an additional filtering process was conducted as specified in SAMTOOLS_VIEW_FILTERS2 ("-q 30") at line 42, focusing on the removal of mappings with quality scores below Q30.

**Fig. 6:**
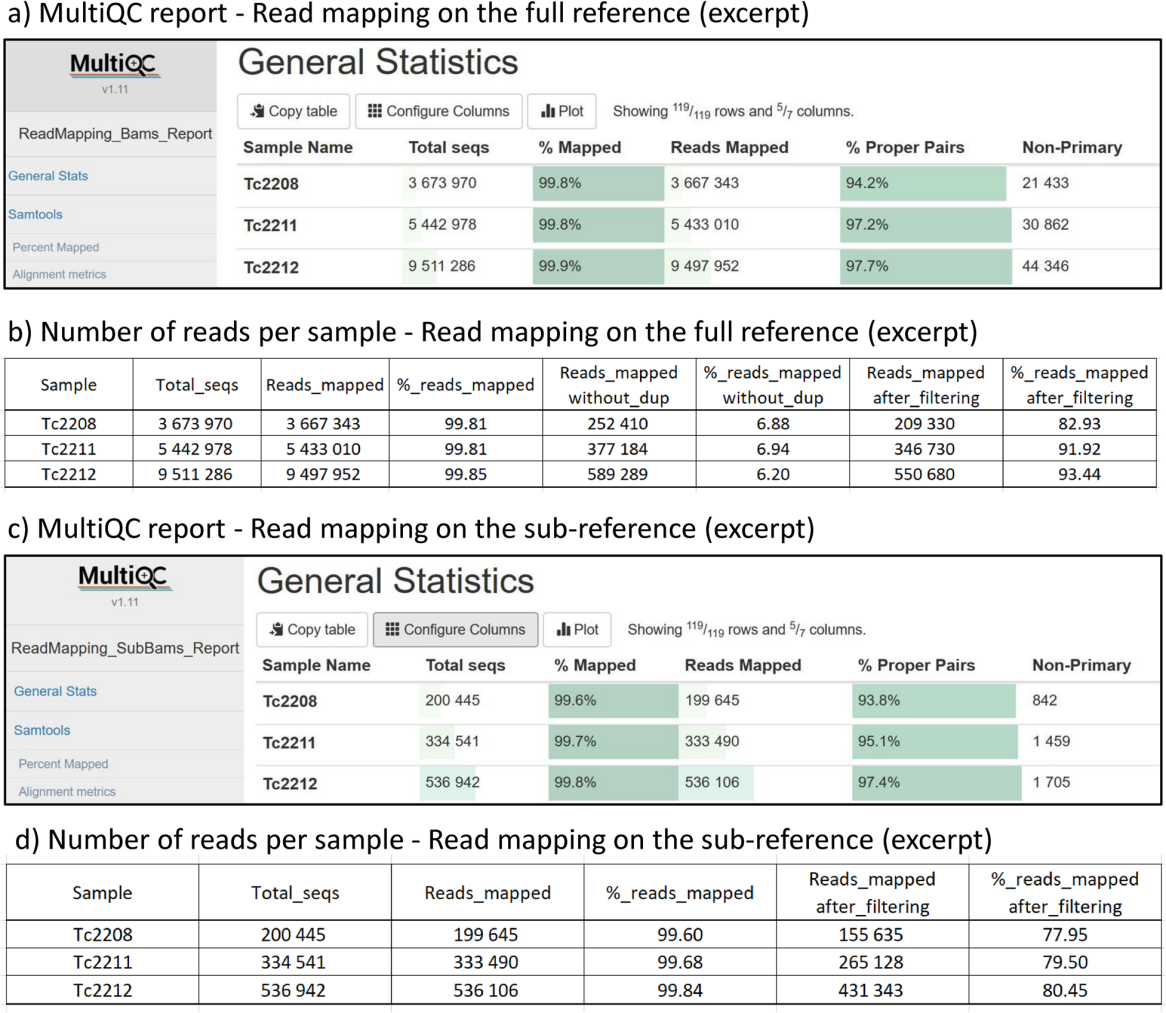
Key outputs of the GeCKO read mapping workflow. The MultiQC report (**a**) and the impact of filtering on read count (**b**) regarding the mapping on the full reference, as well as the MultiQC report (**c**) and the impact of filtering on read count (**d**) regarding the targeted remapping on the sub-reference

The workflow automatically computes various statistics from the raw BAM files, including the percentages of reads that have been successfully mapped and the percentages of properly paired reads. These are summarized in HTML report webpages generated by MultiQC, relevant to both the mapping on the full reference (Fig. 6a) and the targeted remapping on the sub-reference (Fig. 6c). Additionally, the nb_reads_per_sample.tsv files (Fig. 6b and d) provide a detailed view of the impact of the filtering processes applied after each mapping step. For instance, for the individual Tc2208, 99.81% of reads (equivalent to 3,667,353 reads) mapped to the full reference. After excluding PCR duplicates, improperly paired reads, non-primary, and supplementary alignments, 209,330 reads remained. Among these reads, 199,645 mapped to the sub-reference, with 155,635 having a mapping quality greater than Q30.

Lastly, a tabular file named ‘mean_depth_per_zone_per_sample.tsv’ is generated, offering detailed depth coverage information, with each line representing a specific locus and each column a specific sample. This file serves as a useful resource for assessing capture efficiency based on the baits and samples utilized.

#### The VariantCalling workflow

##### Purpose and description

This workflow performs variant discovery and genotyping of samples based on the mapping of the cleaned reads along the reference. The workflow begins with reference indexing, which is skipped if the index files already exist. It then proceeds through a three-phase process: initially, ‘GATK HaplotypeCaller’ is employed to process each sample individually; subsequently, these individual results are merged into a common database using ‘GATK GenomicsDBImport’; this database is then used for variant calling and genotyping the entire population via ‘GATK GenotypeGVCFs’.

A file listing the paths to the multiple BAM files to be used as input (one BAM file per line) should be provided through the VariantCalling config file. If the ReadMapping workflow was used to obtain the BAM files, this file was automatically generated. In addition, this configuration file allows specifying the path to the mapping reference and the parameters for each of the three steps of the GATK variant calling procedure.

If targeted remapping was performed, the variant calling will be significantly sped up. In this case, the workflow will automatically handle the conversion of variant coordinates from the sub-reference back to their corresponding genomic coordinates on the full reference.

##### Use case: variant calling and accession genotyping

**Fig. 7:**
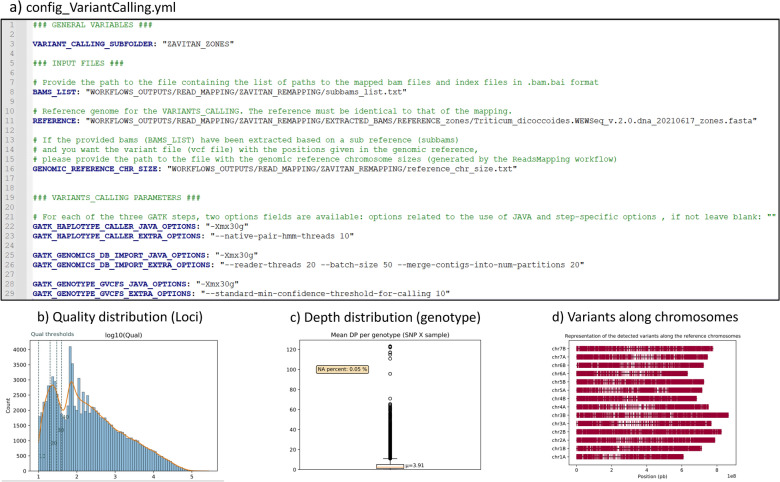
Config file and key outputs of the GeCKO variant calling workflow. The YAML workflow configuration file (**a**), and three key elements of the GeCKO variant calling report files, namely a distribution of the polymorphic site qualities, with dotted lines representing frequently used threshold values (10, 20, 30, and 40) (**b**) a boxplot of the number of reads per predicted genotypes (**c**) and the distribution of the variants along chromosomes (**d**)

Given that the reads have been extracted using a sub-reference with the Read Mapping workflow, the following three parameters must be specified in the config file (Fig. 7a): the list of sub-BAM files (L8), the sub-reference fasta file (L11), and a file detailing the genomic reference chromosome sizes (L16). These files, generated by the GeCKO mapping workflow, are readily available for use in this step. Additionally, the parameters for the three GATK variant calling steps can be adjusted as needed (L22–29).

Three PDF report files are produced: two encompass descriptive statistic plots, one at the locus level (variants_stats_histograms_VC.pdf) and another at the genotype level (genotypes_DP_boxplot_VC.pdf). These plots offer valuable insights, such as the distribution of polymorphism site quality (Fig. 7b) and a box plot of the number of reads per genotype inference (Fig. 7c), which are useful for informing subsequent filtering decisions. The third report (variants_along_genome_VC.pdf) presents a plot of the distribution of the predicted SNPs along chromosomes (Fig. 7d). Three analogous PDF reports are generated by the VcfFiltering workflow, allowing one to visualize the effects of the filtering process.

#### The VcfFiltering workflow

##### Purpose and description

This workflow chains four filtering steps to transform the raw VCF file, produced at the previous step, into a dataset suitable for downstream analysis. It expects a VCF file containing the inferred genotype (cell) for each sample (columns) at each locus (rows), such as produced by the VariantCalling workflow. Some filtering steps mask individual genotypes (replacing them with a missing value), whereas others remove whole samples or loci. Our first filtering step aims to mask the less reliable genotypes with the “filter” command of the BCFtools software. Numerous criteria can be used for this purpose, including the confidence of the variant caller in the genotype prediction (GQ) or the number of reads used for this prediction (DP). The second filtering step, also done using BCFtools, removes the less reliable loci (matrix rows), e.g. those with too many missing values (F_missing), or with poor quality (QUAL). The third filtering step removes samples that introduce too many missing values. This is done using a combination of the “query” and “view” commands of BCFtools together with some awk instructions. The fourth and final step adds some extra population genetic indicators at the SNP level estimated with EggLib after the above described filtering steps. An additional locus-filtering step based on these population genetic indicators can then be applied. Full control of the options passed to BCFtools is provided to the end-user, which can hence fine-tune the filtering process as desired.

##### Use case: filtering the genotyping matrix for genetic analysis to document tetraploid wheat domestication

**Fig. 8:**
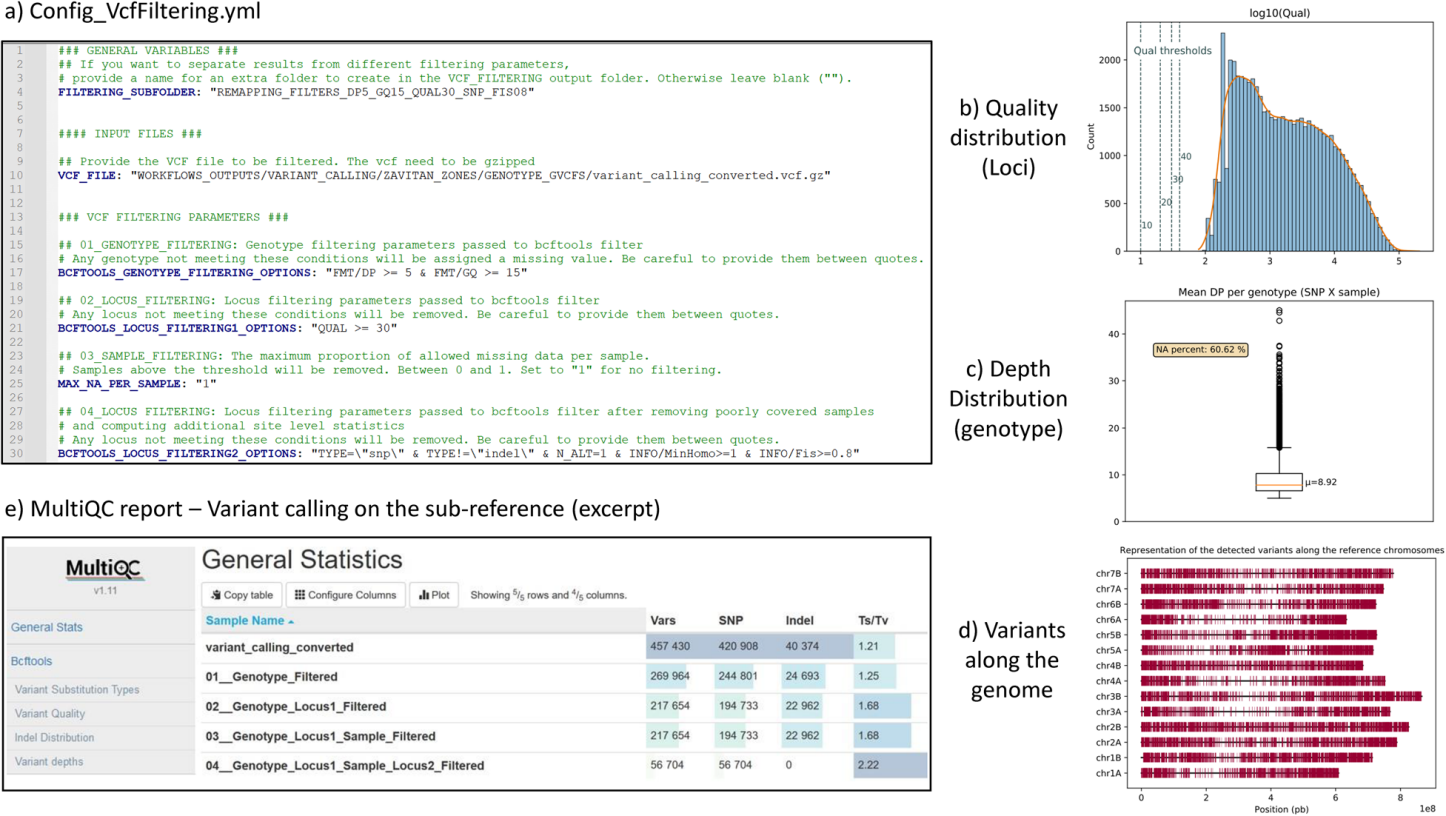
Config file and key outputs of the GeCKO vcf filtering workflow. The YAML workflow configuration file (**a**) and the four key elements of the GeCKO VcfFiltering report files, namely the distribution of the SNP quality (**b**), a boxplot of the number of reads per predicted genotypes (**c**), a plot of the distribution of these SNPs along chromosomes (**d**) and a table provided summary statistics of the four filtering steps (**e**)

The filters applied to the genotype and variant predictions in the GeCKO VcfFiltering workflow are highly dependent on the downstream analyses and prior biological knowledge and can be adjusted based on the resulting output. In our case, we parameterized the four filtering steps, denoted as 01–04 in the config and report files, as shown in Fig. 8a. First, we considered only genotypes predicted by at least 5 reads (being more stringent on this parameter would seem unreasonable according to Fig. 7c) with a GQ greater than 15 (01: L17). Second, we excluded loci with a quality lower than 30 (02: L21). Third, we did not filter samples based on their ratio of missing data (03: L25). Fourth, we focused on SNPs with no more than two alleles. Additional filtering was necessary to discard spurious SNPs commonly found in durum wheat sequencing data, due to its complex polyploid genome. Indeed, reads from different homeologous or paralogous regions can erroneously be mapped to the same locus due to sequence similarities, resulting in spurious SNPs and apparent heterozygosity. Fortunately, the plant’s predominant self-fertilization -with outcrossing rates between 1 and 4% [41]—renders any excess heterozygosity suspicious, thus facilitating the identification of unlikely SNPs. Considering an outcrossing rate (t) of 4%, and using Weir’s formula [42] $FIS=\frac{1 - t}{1 + t}$, we expect FIS values to be distributed around a mean of 0.92. Consequently, and following what was done in Holtz et al. [9], we excluded SNPs with a FIS < 0.8, indicating abnormally high heterozygosity, or those where the minor allele was not observed at least once in the homozygous state, as these were likely to reflect technical artefacts rather than genuine genetic variation (04: L30).

The MultiQC report includes a summary table that details the numbers of SNPs and the corresponding transition/transversion ratio (Ts/Tv) at each filtering step (Fig. 8e). This ratio is a well-established metric for assessing the quality of inferred SNPs [43]. Indeed, in biological systems, transitions (Ts) occur more frequently than transversions (Tv) due to the structural similarities among purines (A, G) and pyrimidines (C, T), typically resulting in a Ts/Tv ratio greater than 2 in high-confidence SNP datasets. In contrast, artefactual SNPs do not adhere to this biological rule. Sequencing errors generally occur randomly, producing false SNPs with a Ts/Tv ratio of 0.5, as there are two transitions and four transversions among all possible substitution types. In datasets where SNPs have not been filtered, the presence of false positives consequently lowers the overall Ts/Tv ratio [43].

In our case, its evolution through the filtering steps confirms the relevance of each filter. Initially, the raw GATK output yielded 420,908 SNPs with a Ts/Tv ratio of 1.21, which is unusually low. The genotype filtering step (01) together with the locus filtering based on GATK quality metrics (02) reduced the SNP count to 197,733 and resulted in a more plausible Ts/Tv ratio of 1.68. The absence of individual/sample filtering (03) meant no change at this step. The final filter (04), targeting bi-allelic SNPs compatible with durum wheat’s autogamous reproduction, further narrowed the SNPs down to 56,704. However, their Ts/Tv ratio of 2.22 aligns considerably better with previous studies (e.g., Ts/Tv around 2.11 in bread wheat [44, 45]), indicating a significant improvement in overall loci quality (Figs. 7b vs 8b) and in mean depth per genotype (Figs. 7c vs 8), while still preserving a sufficient number of SNPs to comprehensively cover the whole durum wheat genome (Figs. 7d vs 8d).

### Use case: validation of the genotypes produced via GeCKO

To illustrate the analysis possibilities of the GeCKO workflow outputs, the VCF file generated at the end of the filtering workflow was used as a starting point to look into the effects of domestication transitions in durum wheat.

#### Population structure consistent with domestication history

**Fig. 9:**
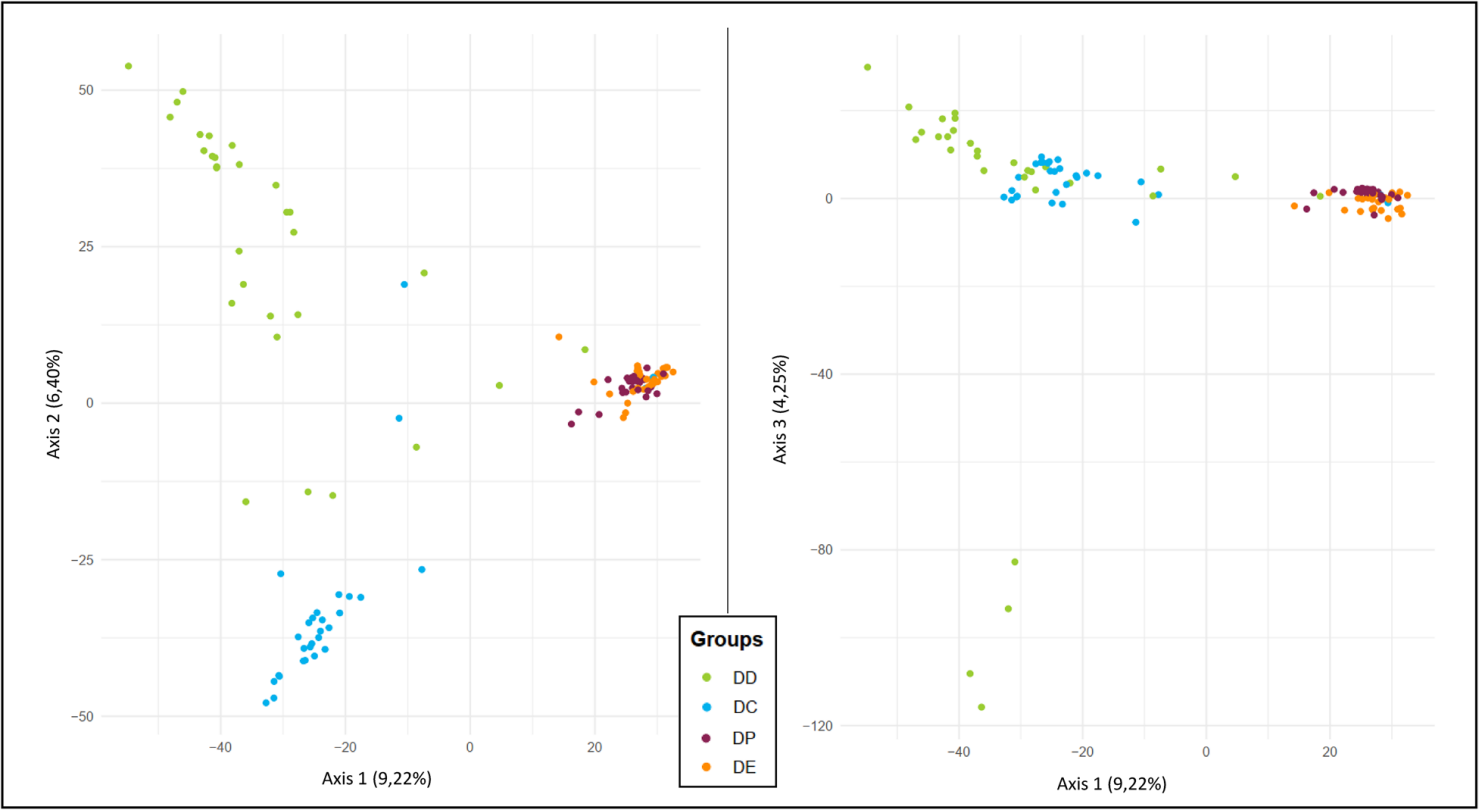
PCA plots (axis 1–2 and 1–3) of the four durum wheat populations (DD, DC, DP, and DE)

We started by exploring the structure of the four populations with a principal component analysis (PCA). For this purpose, we focused on the 9,077 SNPs for which at least 20 individuals per group were genotyped, and the missing genotypes were imputed using the Beagle 5.4 tool [46]. After imputation, the PCA (Fig. 9) was performed with the R adegenet and ade4 packages [47]. The first three axes of the PCA accounted for 19.9% of the total variance. The first axis of the PCA, accounting for 9.22% of the total variance, seems to reflect the complete domestication process from the wild form (DD) to the more recent forms of durum wheat DP and DE. The second axis, explaining 6.40% of the variance, appears to mainly represent the differentiation between the DD and DC groups. The third axis (4.25%) essentially captures the diversity within the DD group, with four DD individuals (bottom left) deviating significantly from all other samples.

Additionally, pairwise Fst values, a standard estimate of population divergence based on allelic frequencies, were computed. At the genome level, the divergence is mainly driven by population size (gene drift) and isolation (gene flow). Focusing on the 25,174 SNPs that had at least 10 individuals genotyped per population, we used EggLib to estimate Fst values for each transition: Fst_DD-DC_ = 0.230, Fst_DC-DP_ = 0.348, and Fst_DP-DE_ = 0.135. As expected, each transition’s impact on allelic frequencies resulted in a notable divergence between populations (Fst > 0). Our data indicate that the most substantial differentiation (Fst = 0.348) occurred during the transition to naked grains (from DC to DP).

#### Diversity loss during the domestication process

**Fig. 10:**
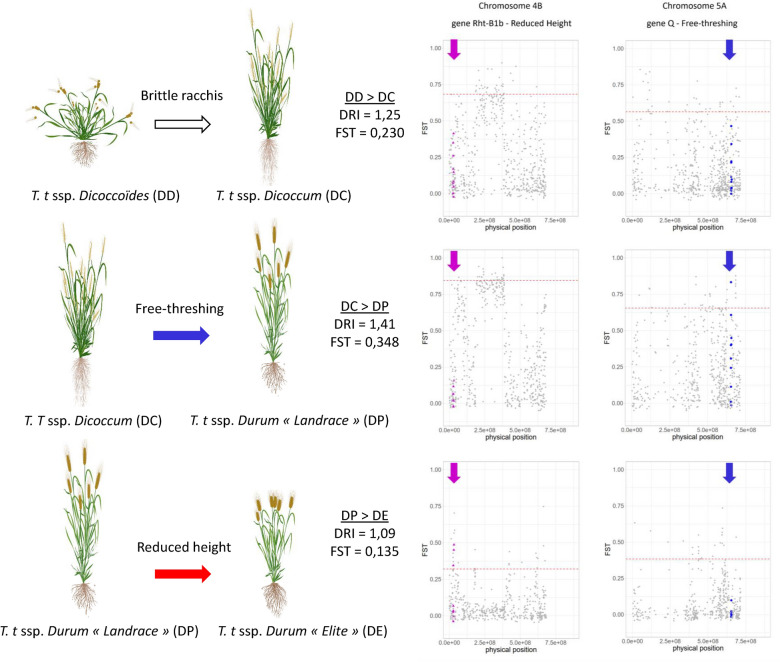
DRI and Fst values for the three transitions and Fst-scan analysis of chromosomes 4B and 5A. The Rht-B1b gene is known to be involved in the height reduction of plants during the DP to DC transitions. Magenta triangles represent loci nearby Rht-B1b and magenta arrows indicate the corresponding genomic region. Likewise, blue dots represent loci nearby gene Q, which is known to be involved in the shift toward free-threshing, during the DC to DP transition; blue arrows pinpoint the corresponding genomic region. Red lines represent the threshold for Fst-values significantly higher than the chromosome average (95% of Fst-values below this line)

We then further investigated the influence of the successive domestication transitions on allelic diversity. The bottleneck implied by a domestication process is known to result in an allelic diversity decrease. To estimate the allelic diversity of each population, we used the same 25,174 SNPs as previously and relied on EggLib to compute the nucleotide diversity (pi). The Diversity Reduction Index (DRI) was then calculated for each domestication transition as the ratio between the allelic diversity of the two populations framing this transition; the higher this value, the stronger the loss of diversity (Fig. 10). Each transition, as expected, results in a decrease in allelic diversity. Our data suggest that the greatest loss of diversity (DRI = 1.41) occurred during the transition to naked grains (from DC to DP). This result is in agreement with the much broader analysis conducted by Maccaferri et al. [12].

#### Selection footprints found at known domestication genes

Additionally, in order to investigate gene-scale selection effects, we conducted pairwise Fst-scan analyses on SNPs with at least 10 individuals genotyped per population for each pair, using EggLib. Fst-scan analysis is based on the expectation that loci under positive selection, along with those in close linkage disequilibrium, will exhibit higher Fst values compared to the genomic background. A common way to identify these loci is to select the ones above the 95% quantile of the distribution of Fst values across the whole chromosome. In our study, we focused on two genes involved in the domestication process: the gene *Q* (chr. 5A) which, among other functions, plays a crucial role in the free-threshing trait that distinguishes the transition between *T. turgidum dicoccoides* and *T. turgidum durum* [48] and the gene *Rht-B1b* (chr. 4B) which was intensively used during the green revolution to develop elite modern cultivars to reduce plant height [49].

The Fig. 10 shows the Fst-scan for chromosomes 4B and 5A for the three domestication transitions. Fst values were computed per zone, identified during the sub-reference creation process. The zones within 5 Mb around the genes of interest were highlighted in magenta (*Rht-B1b*, triangular shape) and blue (*Q*, dots). On each graph, the 95% quantile was represented by a dotted red line, and loci positioned above this threshold were thus considered to be potentially under positive selection.

When comparing DC and DP populations, a peak in Fst-values was observed near gene *Q* (blue dots in Fig. 10); in addition, when comparing DP and DE populations, a strong Fst signal was obtained in the gene *Rht-B1b* region (magenta triangles in Fig. 10). This signal is all the more convincing as it does not appear at other transitions. This strong Fst signature was expected since free-threshing and reduced height were intensively selected during these two domestication transitions respectively.

## Discussion

Our study, employing target enrichment capture to explore durum wheat's genetic diversity, yielded consistent results across various analyses. The PCA differentiated the DD and DC populations, in agreement with their high Fst value (0.230), yet it was unable to distinctly separate the DP and DE populations, consistent with their low Fst value (0.135) and minor diversity difference (DRI = 1.09). These findings align with both the theoretical expectations for this dataset and the results of previous studies [12]. Overall, it confirms the suitability of enrichment capture for genotyping multiple individuals from species with complex genomes, such as durum wheat.

In this case study, GeCKO efficiently streamlined the processing of numerous multiplexed samples, transforming raw sequenced data into a genotyping matrix with just four commands. It also significantly improved the computational performance through its targeted remapping feature, consisting of extracting targeted reads, creating a sub-reference representing just 0.1% of the 10.5 Gb whole reference, and subsequently remapping these reads onto the sub-reference.

This feature not only speeds up processing times, but also enables the analysis of extensive genomes that are beyond the capabilities of some bioinformatics tools such as GATK.

The existence of a tool like GeCKO, specifically tailored for target enrichment capture data, paves the way for the wider application of this method in a variety of studies. This could be particularly beneficial for advancing genomic research in species with notably complex or large genomes.

In the development of GeCKO, a strong emphasis was placed on adhering to the FAIR principles. As a result, GeCKO is readily *findable* and *accessible*, being hosted on GitHub, which provides it with a unique identifier and simplifies its acquisition through cloning. Its *interoperability* is ensured by compliance with POSIX standards and use of widely-used genomic file formats like BAM and VCF, facilitating its integration with other genomic tools. Additionally, GeCKO’s *reusability* is supported by comprehensive documentation, including dataset and command lines for step by step examples and a full-scale use case. Finally, its automated reporting mechanism, meticulously logging tool versions and settings as well the date, time and GeCKO commit ID of each run, guarantees thorough documentation of every analysis step. This feature, operating seamlessly in the background, advances both transparency and reproducibility effortlessly for the user.

GeCKO was designed to be user-friendly, aiming to be accessible to geneticists with basic bioinformatics skills. It simplifies various genomic analysis tasks, handling file format conversions, software tool execution, error management, and sending job requests to execute tasks on High-Performance Computing systems. It also generates detailed reports after each run, facilitating rigorous monitoring and quality assessment during the analysis. Yet, despite its automated functions, GeCKO still maintains a high degree of flexibility, allowing it to accommodate a broad spectrum of tasks (from processing whole genome sequencing to genomic reduction data). Its structure offers the option to chain the four workflows sequentially or use them independently, depending on the user’s requirements. Additionally, the configuration files grant users the possibility to fine-tune parameters and options for the software tools called in the workflows. It also offers a wide range of options, such as processing either paired-end or single-end data, excluding unnecessary steps like demultiplexing or targeted remapping, or selecting from a variety of mappers for the read mapping step.

## Conclusion

GeCKO (Genotyping Complexity Knocked-Out)’s main achievements, demonstrated with a case study on durum wheat domestication, include streamlining the genotyping process and improving computational efficiency for processing target enrichment capture data through innovative features like targeted remapping. The tool’s adherence to the FAIR principles ensures it is findable, accessible, interoperable, and reusable, promoting transparency and reproducibility in genomic research. Furthermore, GeCKO’s user-friendly design, catering to geneticists with basic bioinformatics skills, and its flexibility in handling a variety of genomic analysis tasks, broaden the accessibility of genotyping and studying species with large and complex genomes.

While GeCKO already offers a diverse set of functionalities, we are committed to its continuous improvement and expansion for the exploitation of genome reduction datasets. A significant future development will be a bait design workflow for target enrichment capture, which will help researchers to design their own bait set for their genomic studies. Additionally, we plan to add a new feature to the Data Cleaning workflow to accommodate libraries created with Unique Molecular Identifiers (UMIs). This will allow to accurately identify and remove true duplicates, and help address the challenges associated with high rates of duplicates in sequencing data. Importantly, this feature would enable the processing of data generated with Molecular Inversion Probes (MIPs), an effective alternative to target enrichment capture for genome reduction. In the context of MIPs technology, which utilizes targeted probes to amplify specific genomic regions, the differentiation between PCR duplicates and authentic reads—originating from identical sequences but different molecules—is crucial. The use of UMIs then becomes indispensable, making their integration into GeCKO a valuable addition.

## Data Availability

The GeCKO workflows are freely available on a dedicated GitHub repository (https://github.com/GE2POP/GeCKO). The dataset supporting the conclusions of this article is available in the French dataverse repository https://entrepot.recherche.data.gouv.fr/ (10.57745/78MBZY). All scripts needed to reproduce these analyses are provided on a GitHub repository (https://github.com/GE2POP/GeCKO_UseCase).
